# Beetroot juice supplementation enhances the effects of blood flow restriction training on lower limb strength and vertical jump performance under fatigue in male university students: a randomized, double-blind, placebo-controlled study

**DOI:** 10.1080/15502783.2026.2636613

**Published:** 2026-02-24

**Authors:** Xudong Yang, Yue Lu, Sang Ki Lee, Hongqi Xu, Hualong Chang, Qing Liu, Helong Quan

**Affiliations:** a Research Center of Exercise Capacity Assessment and Promotion, School of Physical Education, Northeast Normal University, Changchun, Jilin, People's Republic of China; b Department of Sport Science, Chungnam National University, Daejeon, South Korea; c Faculty of Sports Science, Ningbo University, Ningbo, People's Republic of China; d Jilin Green Food Engineering Research Institute, Changchun, Jilin, People's Republic of China

**Keywords:** Blood flow restriction, beetroot juice, countermovement jump, muscle strength, fatigue

## Abstract

**Background:**

Beetroot juice (BRJ) supplementation has the potential to enhance the effects of blood flow restriction (BFR) training in improving muscle strength and fatigue resistance; however, evidence supporting their combined effects remains limited. This study investigated whether BRJ supplementation enhances the effects of BFR training on muscle strength and fatigue resistance.

**Methods:**

This randomized, double-blind, placebo-controlled trial included 20 male university students from the School of Sports, who were randomly assigned to a BFR group (*n* = 10, age: 21.9 ± 1.7 years) or a BFR + BRJ group (*n* = 10, age: 21.8 ± 1.5 years; nitrate: 8 mmol/day). Participants completed a one-week BRJ pre-supplementation phase followed by a four-week bilateral knee extensor/flexor BFR training program (40% limb occlusion pressure, 30% of peak torque load), performed three times per week. Knee extensor and flexor strength (at 60°/s, 180°/s, and MVIC) and countermovement jump (CMJ) performance were assessed using an isokinetic dynamometer and a force plate pre- and post-intervention.

**Results:**

Both the four-week BFR and BFR+BRJ interventions significantly increased the relative peak torque of the knee extensors/flexors at 60°/s (Left: *p*_*pre-post*_ = 0.23, d = –0.89, ηp²_*Time*_ = 0.775; Right: *p*_*pre-post*_ = 0.017, d = –0.63, ηp²_*Time*_ = 0.744), 180°/s (Left: *p*_*pre-post*_ = 0.028, d = –1.32, ηp²_*Time*_ = 0.319; Right: *p*_*pre-post*_ = 0.007, d = –1.48, ηp²_*Time*_ = 0.822), and MVIC (Left: *p*_*pre-post*_ = 0.007, d = –0.11, ηp²_*Time*_ = 0.825; Right: *p*_*pre-post*_ = 0.009, d = –1.31, ηp²_*Time*_ = 0.842). They also improved the torque of the knee extensors in both the left and right legs during the 100-repetition maximal voluntary contraction test at 90°/s, with both initial (first 20 reps) and final (last 20 reps) values significantly increased (Left: *p*_*pre-post*_ = 0.029, d = –0.96, ηp²_*Time*_ = 0.612; Right: *p*_*pre-post*_ = 0.007, d = –1.21, ηp²_*Time*_ = 0.725). The CMJ test showed significant improvements in fatigued bilateral CMJ height (*p*_*pre-post*_ = 0.048, d = –0.534, ηp²_*Time*_ = 0.556), peak force (*p*_*pre-post*_ = 0.047, d = –0.913, ηp²_*Time*_ = 0.444), and rate of force development (RFD; *p*_*pre-post*_ = 0.044, d = –0.902, ηp²_*Time*_ = 0.656) following both BFR and BFR + BRJ interventions. Notably, single-leg countermovement jump performance showed no significant improvements for either the left or right leg. Notably, post-intervention, only the BFR+BRJ group showed significant improvements in fatigued bilateral CMJ height (*p*_*pre-post*_ = 0.012, d = –1.307, ηp²_*Time*_ = 0.846).

**Conclusions:**

The results indicate that four weeks of BFR training, with or without BRJ supplementation, can improve knee flexor and extensor strength and bilateral CMJ performance. However, the effects of BRJ were selective rather than broadly superior, as BRJ mainly enhanced BFR training by reducing fatigue-related declines in vertical jump performance.

## 1 Introduction

With increasing high-intensity intermittent loads and biomechanical challenges in modern competitive sports, lower limb strength (particularly the knee flexor/extensor muscles' isometric and concentric strength capacity and eccentric-concentric transition efficiency) and fatigue resistance (including energy metabolism, metabolite accumulation, and neuromuscular fatigue) are crucial for sustaining repeated jump performance and injury prevention [1]. In sports requiring frequent jumping (volleyball, basketball, soccer, and rugby), continuous jumping causes lower-limb neuromuscular fatigue, thereby affecting athletes’ performance and results [2,3]. Notably, fatigue-induced declines in knee joint strength result in a reduced knee flexion angle and increased valgus moment during landing [4,5], which may increase the risk of sustaining non-contact lower limb injuries (e.g. rupture of the anterior cruciate ligament) [6,7]. In individual and team sports, countermovement jump (CMJ) tests assessing flight time (FT), jump height (JH), and rate of force development (RFD) are widely used to monitor lower-limb neuromuscular status [8]. CMJ serves as an indirect indicator of explosive lower-body power and strength across various sports [9–11]. Therefore, enhancing knee flexor/extensor strength and fatigue resistance is a key intervention target for optimizing jump performance and preventing injuries [12,13]. To further improve these outcomes, high-efficiency training methods combined with nutritional strategies like nitrate supplementation have received increasing attention.

Blood flow restriction (BFR) training partially restricts venous return and arterial inflow, inducing localized muscle hypoxia, and has recently attracted attention in sports and rehabilitation for its ability to promote muscle hypertrophy and strength at low loads. This environment increases skeletal muscle metabolic stress, thereby promoting cardiovascular adaptations [14], hormonal responses [15], and muscle oxidative capacity [16], and stimulating protein synthesis [17] and activating the myogenic stem cell pathway [18] to improve muscle structure and function. In practice, BFR training enhances muscle strength and hypertrophy across athletic populations, including college-aged individuals, and positively influences performance and fatigue resistance [19]. Notably, several studies have explored the effects of BFR on countermovement jump (CMJ) performance. Sun et al. reported that low-intensity BFR (50%−60% arterial occlusion pressure, AOP) combined with half-squat training increased CMJ height, peak power, and RFD development via enhanced muscle activation and post-activation potentiation (PAP) [20], while high-pressure BFR (200 mmHg) was ineffective due to suppressed adaptation [21]. Compared to traditional high-load resistance training, low- to moderate-intensity BFR protocols produced a similar PAP effect while prolonging its duration, thus improving CMJ performance [22]. Furthermore, BFR during post-exercise recovery (80% AOP) accelerated CMJ height recovery (48-hour difference of –2.8 cm, *p* = 0.019) and reduced muscle damage [23]. Moderate BFR (50–60% AOP) has been shown to be a viable strategy to optimize CMJ performance. Despite these benefits, BFR alone may have limited effects on muscle endurance and metabolic adaptations [24,25]. Recent studies suggest that combining BFR with dietary nitrate supplementation, such as beetroot juice (BRJ), could further enhance muscle performance by improving nitric oxide availability, blood flow, and fatigue resistance [24,25]. This combination provides a novel approach for exploring the synergistic effects on muscle endurance and athletic performance.

Beetroot juice (BRJ) is widely used to enhance performance, mitigate fatigue, and facilitate post-exercise recovery. BRJ contains high levels of betaine [26] and nitrate [27,28]. Betaine exhibits potent antioxidant and anti-inflammatory properties [29] that effectively neutralize reactive oxygen species (ROS) produced during intense exercise [30]. This, in turn, alleviates inflammation associated with muscle fiber damage and accelerates recovery. The nitrate in BRJ is converted to nitric oxide (NO) via the nitrate–nitrite–NO pathway, which facilitates vasodilation and enhances the delivery of oxygen, hormones, and nutrients to active muscles. Notably, chronic BRJ supplementation has been demonstrated to enhance neuromuscular function by facilitating calcium ion (Ca²⁺) release from the sarcoplasmic reticulum [31], optimizing excitation-contraction coupling [32], and attenuating the development of neuromuscular fatigue [33,34]. While dietary nitrate supplementation (e.g. BRJ) has been shown to enhance muscle contractility [30] and fatigue resistance [27], its isolated impact on countermovement jump (CMJ) performance may be condition- and gender-dependent. In acute studies, a single high-dose intake of nitrate (BRJ, approximately 12.8 mmol NO₃^–^) significantly increased plasma nitrate and nitrite levels (*P* < 0.001) but did not improve CMJ height or post-jump recovery in male rugby players [35]. Similarly, healthy active males showed no significant change in CMJ height after 6 days of BRJ supplementation (985 mg/d) [36]. However, in female populations, acute BRJ supplementation (400 mg nitrate) significantly improved maximal CMJ height and lower-limb explosiveness (mean power increased by 7.3% at 50% 1RM, *P* = 0.02) and enhanced muscular endurance (increased repetitions, *P* < 0.001) [37]. Additionally, in female volleyball players, BRJ did not directly improve CMJ height but indirectly supported recovery by reducing muscle swelling and perceived soreness [38]. These findings suggest potential sex-specific responsiveness, as the more consistent performance benefits have been observed in female participants. However, evidence in males remains limited, and further research is required to clarify optimal dosing strategies and the magnitude of BRJ’s effects in male populations. Overall, the benefits of BRJ on CMJ performance may require combination with strength or sport-specific training.

Although BFR and BRJ improve muscle strength and fatigue resistance through distinct physiological mechanisms, the local hypoxia induced by BFR coupled with the nitrate-mediated enhancement of blood flow and improved neuromuscular function may produce a synergistic effect on skeletal muscle strength and performance. The individual effects of BFR training and BRJ supplementation have been extensively investigated, while evidence regarding their combined benefits remains limited. This study aims to investigate whether the combination of BFR training and BRJ supplementation more effectively enhances knee extensor-flexor strength and as well as both non-fatigued and fatigued CMJ performance compared to BFR training alone.

## 2 Methods

### 2.1 Participants

Twenty male undergraduate students from the School of Sports voluntarily participated in this study. All participants were free of chronic diseases, including cardiovascular conditions, heart disease, diabetes, and other metabolic disorders; had not used nitrate supplements in the past six months; and had no history of musculoskeletal injuries or surgery. They were instructed to avoid using mouthwash (to prevent interference with nitrate reduction), sports supplements, alcohol, and caffeine throughout the study. After the study protocol were explained, all participants provided written informed consent. The study adhered to the Declaration of Helsinki and received approval from the Ethics Committee of Northeast Normal University (Approval ID: 20242026).

### 2.2 Study design

**Figure 1.**
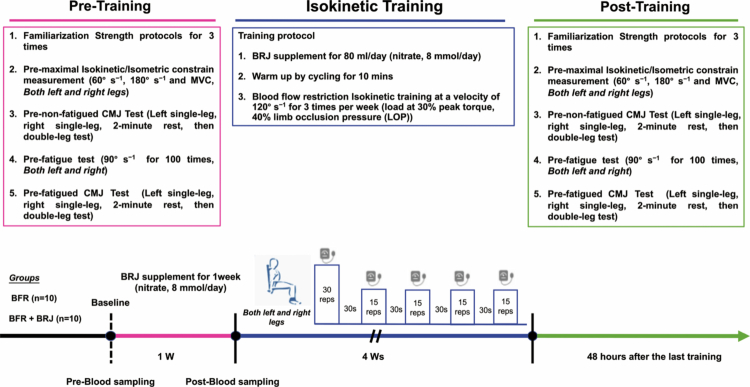
Schematic representation of the study design.

This study was conducted as a randomized, double-blind, placebo-controlled trial, comprising a one-week BRJ pre-supplementation phase followed by a four-week bilateral knee extensor/flexor BFR training program (three sessions per week). Participants were stratified by body weight in 5-kg increments, starting from 65 kg, and then randomly assigned into six blocks to either the BFR group (*n* = 10, age 21.9 ± 1.70 years, body weight 76.6 ± 6.02 kg, height 1.798 ± 0.04 m, BMI 23.67 ± 1.23 kg·m^−2^; mean ± SD) or the BFR + BRJ group (*n* = 10, age 21.8 ± 1.5 years, body weight 77.1 ± 6.56 kg, height 1.795 ± 0.05 m, BMI 24.24 ± 2.10 kg·m^−2^; mean ± SD). A random allocation sequence was generated and maintained by two independent coordinators who were not involved in recruitment, training supervision, data collection, or outcome assessment. They prepared the coded supplements to ensure full allocation concealment. During the supplementation, training, and testing phases, two independent researchers were responsible for data collection, and both the participants and assessors were blinded to group allocation and supplement content. Following three familiarization sessions with laboratory procedures, participants underwent baseline testing of knee extensor-flexor strength using the CON-TREX multi-joint isokinetic system (CON-TREX® MJ, Physiomed Elektromedizin AG, Schnaittach, Germany), and countermovement Jump (CMJ) performance using the AMTI Force Plate (BMS600900, Advanced Mechanical Technology, Watertown, USA) prior to the intervention. These tests were repeated after the four-week intervention to assess post-intervention knee extensor-flexor strength and CMJ performance. The detailed experimental protocol is shown in Figure 1.

### 2.3 Beet juice supplementation strategies

The nitrate supplementation strategy was based on the optimal dose recommended in previous studies [39,40]. The BFR+BRJ group ingested 80 mL of BRJ (nitrate 8 mmol/day, M-ACTION, Shanghai, China) daily throughout the study, while the BFR group consumed an identical dose of placebo (PLA, a mixture of food coloring and commercially available purple sweet potato juice). Both BRJ and PLA were stored in opaque, unlabeled glass containers to ensure effective blinding. Participants consumed the supplement at 14:00–17:00 during the pre-supplementation phase and on non-training days (except for the final pre-supplementation dose prior to blood samples collection), and two hours before training sessions on training day [39]. No participants reported or exhibited any adverse effects throughout the study.

### 2.4 Blood collection and analysis

At both the start and end of the pre-supplementation phase, blood samples (10 mL) were collected in the morning from anticoagulant-treated tubes, approximately 8–12 hours after the last supplement ingestion and prior to any training session. The samples were then centrifuged at 4 °C (2000 × g, 10 minutes) and the plasma nitrite levels were measured using a nitrite colorimetric assay kit (E-BC-K070-S, Elabscience, Wuhan, China) following the manufacturer’s protocol.

### 2.5 Isokinetic training protocol

During the formal training phase, both the BFR and BFR+BRJ groups performed three isokinetic BFR training sessions per week. The isokinetic training was set at 120°/s, with the load on the knee extensor-flexor muscles at 30% of peak torque (peak torque was measured with the CON-TREX system in each session) [41,42]. A blood flow restriction cuff (Thera Tools Cuff 110 × 13 cm, Zhengzhou, China) was applied to induce 40% limb occlusion pressure (LOP). This pressure was selected based on previous studies while adjusted in our study to minimize participant discomfort and pain, thereby reducing potential risks during training, while maintaining an effective BFR stimulus [41,42]. Thigh occlusion pressure (mmHg) was calculated using the following equation = 5.893 (Thigh circumference) +0.734 Diastolic Blood Pressure (DBP) +0.912 Systolic Blood Pressure (SBP) − 220.046, based on Jeremy *P* et al [43]. and validated by Medrano et al. [44]. Each session consisted of five sets (1 × 30 and 4 × 15 reps) with 30-second rest intervals. This protocol was adapted from previous studies and has been used in several investigations with good results [43,44].

### 2.6 Maximum strength and fatigue test protocols

Prior to testing, participants completed a 10-minute warm-up on a stationary bike. Non-fatigue peak torque was assessed using the CON-TREX system at angular velocities of 60°/s and 180°/s (five repetitions per velocity), with a 5-minute rest between tests. Maximum voluntary isometric contraction (MVIC) was evaluated at a fixed knee angle of 75° with a 5-second hold. During testing, the trunk-thigh angle was set to 110°, and the range of motion was individualized based on participant flexibility (0°−90°, where 0° represents full knee extension). Peak torque was defined as the highest knee flexor and extensor moment (*N*) achieved across the five contractions, and average peak torque was calculated as the mean maximal value over the five repetitions.

Following the non-fatigue peak torque tests, participants underwent a 100-repetition maximal dynamic knee extension protocol to elicit knee flexor and extensor fatigue [45]. All repetitions were performed without BFR, at an angular velocity of 90°/s, with maximal effort during both the extension (90°–0°) and flexion (0°–90°) phases [46]. Average torque decline rate was defined as the difference between the average torque of the initial 20 repetitions and the final 20 repetitions, divided by the average torque of the initial 20 repetitions. All participants completed the test on both legs, with the dominant leg defined as the kicking leg.

### 2.7 Movement jump test

The CMJ test involved three conditions: left-leg single-leg jump, right-leg single-leg jump, and two-leg jump. After completing the single-leg tests, participants rested for two minutes before performing the two-leg jump. For each condition, participants crossed their arms over their shoulders and stood motionless on the AMTI Force Plate. Upon receiving the start signal, participants executed a rapid countermovement to their self-selected depth before performing a maximal vertical jump. Each condition was repeated five times. During flight, participants were instructed to maintain full knee extension and refrain from tucking their knees before landing. Ground reaction force (GRF) data were processed with a fourth-order Butterworth low-pass filter (cutoff frequency: 30 Hz). Peak force, jump height, and eccentric rate of force development (RFD) were subsequently calculated. Jump height was determined based on a previous study [47] using the formula: h = (1/8) × g × t², where g represents gravitational acceleration (9.81 m·s⁻²), and t is flight time. Flight time was defined as the duration between take-off (GRF below 5 *N*) and landing (GRF exceeding 10 *N*). Eccentric RFD was calculated using the formula proposed by Barker et al [9].: RFD = (first GRF peak - minimum GRF)/(time), where time represents the time between peak force and minimum force. Eccentric RFD reflects the capacity for explosive force generation and contributes to improved muscle elasticity and energy storage efficiency during the eccentric phase of movement.

### 2.8 Statistical analysis

A priori power analysis was performed using G*Power 3.1 (Heinrich-Heine-Universität Düsseldorf, Düsseldorf, Germany) to determine the required sample size for a repeated-measures ANOVA with a within–between interaction. The analysis assumed a medium effect size (f = 0.4), an *α* level of 0.05, and a desired power of 0.9, resulting in a minimum total sample size of 20 participants. The data were analyzed using GraphPad Prism 9 (GraphPad Software, San Diego, CA, USA). All results are expressed as mean ± standard deviation (SD). Between-group differences in baseline characteristics were analyzed using an independent samples t-test. A two-way repeated measures ANOVA was conducted to evaluate plasma nitrite, jump height, RFD, peak force in CMJ, and (relative peak torque and relative average peak torque at 60°/s and 180°/s, as well as initial/final 20-repetition average torque and average torque decline rate in the 90°/s 100-repetition test). The model included group (BFR vs. BFR + BRJ) and time (pre vs. post) as within-subject factors. A three-way repeated measures ANOVA with factors group (BFR vs. BFR+BRJ), fatigue state (non-fatigued vs. fatigued), and time (pre vs. post) was used to assess jump height, RFD, and peak force in CMJ. Prior to ANOVA, data were checked for normality using the Shapiro-Wilk test. Sphericity was tested using Mauchly’s test, and the Greenhouse-Geisser correction was applied if the assumption was violated. Effect sizes for main and interaction effects were determined using partial eta-squared (ηp²) classified as small (ηp² ≥ 0.01), medium (ηp² ≥ 0.06), and large (ηp² ≥ 0.14). Post hoc analyses were performed using Bonferroni's multiple comparison test when significant main or interaction effects were detected. Statistical significance was set at *p* < 0.05.

## 3 Results

### 3.1 Descriptive characteristics of variables between BFR and BFR+BRJ groups

**Figure 2.**
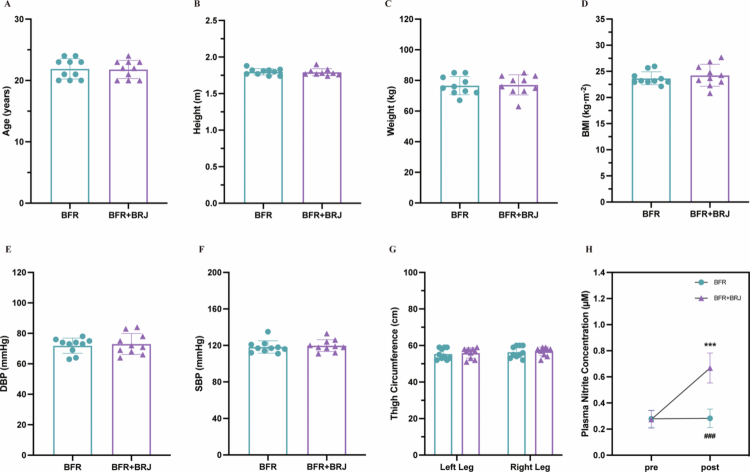
Descriptive Characteristics and Comparison of Variables Between BFR and BFR+BRJ Groups. Values are presented as mean ± standard deviation (SD). BMI, body mass index; DBP, diastolic blood pressure, SBP, systolic blood pressure. Statistical significance is indicated as follows: ^*^ indicates significant difference between post-BFR+BRJ and pre-BFR+BRJ; ^#^ indicates significant difference between post-BFR and post-BFR+BRJ.

Before the intervention, there were no significant differences between the BFR and BFR+BRJ groups in age, height, weight, BMI, resting DBP and SBP, or left/right thigh circumference (Figure 2A–G). Following one week of nitrate supplementation, plasma nitrite levels in the BFR+BRJ group increased significantly, with an absolute rise of 0.39 µmol/L (*p* < 0.001, 95% CI [–0.57, –0.21], Figure 2H). Moreover, post-supplementation nitrite levels in the BFR+BRJ group were also significantly higher than those in the BFR group (*p* < 0.001, 95% CI [−0.55, −0.22]).

### 3.2 Relative peak torque for knee extensors and flexors at pre- and post-intervention

**Figure 3.**
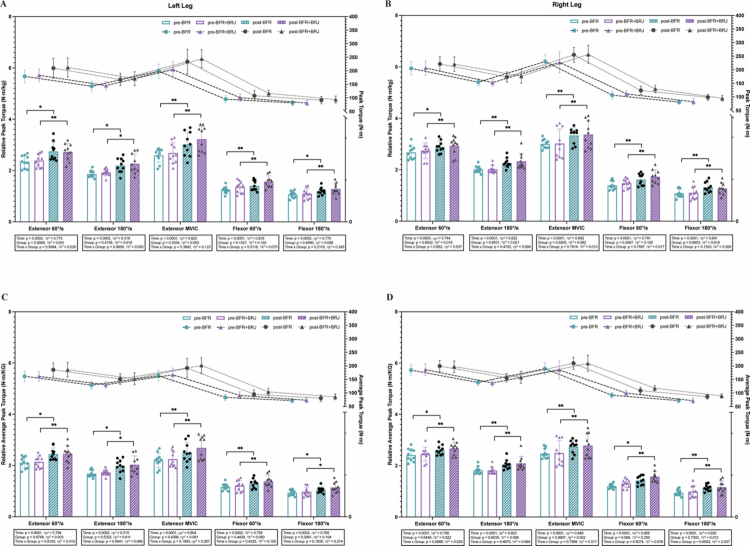
Pre- and Post-Intervention Comparison of Peak Torque, Relative Peak Torque, Average Peak Torque, and Average Relative Peak Torque for Knee Extensors and Flexors under Various Testing Conditions. Values are presented as mean ± standard deviation (SD). Statistical analysis was conducted using a two-way repeated-measures ANOVA (group × time) with Bonferroni's multiple comparisons test for post hoc analysis. Main effects for time (pre vs. post) were statistically significant, while no significant interaction effects (group and group × time) were detected. ηp²: partial eta squared. Significant differences between groups indicated by ^*^P < 0.05 and ^**^P < 0.01.

Relative peak torque increased significantly over time (*p* < 0.001, partial η² > 0.14) in both the BFR and BFR+BRJ groups for the left and right legs (Figure 3A,B), indicating post-intervention improvements. Post-intervention, relative peak torque significantly increased for knee extensors at 60°/s in both groups (BFR: *p* < 0.05, left 95% CI [–0.75, –0.06], right 95% CI [–0.42, –0.04]; BFR+BRJ: *p* < 0.01, left 95% CI [–0.58, –0.09], right 95% CI [–0.34, –0.06]). At 180°/s and MVIC tests, torque also increased significantly across conditions (*p* < 0.05 and *p* < 0.01, respectively). Relative peak torque for the flexors also increased significantly at 60°/s (*P* < 0.01 across conditions; BFR: left 95% CI [–0.29, –0.04], right 95% CI [–0.38, –0.08]; BFR+BRJ: left 95% CI [–0.36, –0.06], right 95% CI [–0.42, –0.07]) and 180°/s (BFR: *p* < 0.05, left 95% CI [–0.23, –0.02], right 95% CI [–0.27, –0.04]; BFR+BRJ: *p* < 0.01, left 95% CI [–0.30, –0.07], right 95% CI [–0.26, –0.08]). Similarly, relative average peak torque in both legs (Figure 3C,D) showed a significant main effect of time (*p* < 0.001, partial η² > 0.14). Post-intervention, both flexors and extensors exhibited consistent increases across 60°/s, 180°/s, and MVIC tests, all significant at *p* < 0.05 across conditions. These findings demonstrate that both BFR+BRJ and BFR interventions effectively enhanced knee flexor and extensor strength, with BFR+BRJ producing a more pronounced improvement in extensor strength at 60°/s.

### 3.3 Quantitative results of jump height, force, and rate of force development (RFD) in CMJ Testing

**Figure 4.**
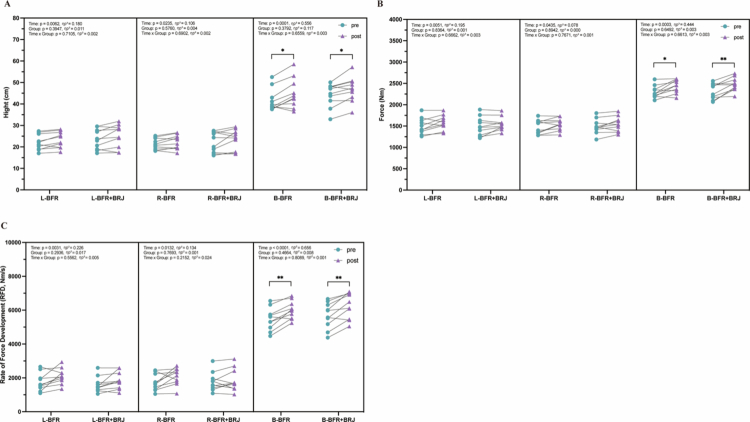
Comparison of Pre- and Post-Intervention Effects of BFR and BFR+BRJ on Movement Jump Test. Values are presented as mean ± standard deviation (SD). Statistical analysis was conducted using a two-way repeated-measures ANOVA (group × time) with Bonferroni's multiple comparisons test for post hoc analysis. Main effects for time (pre vs. post) were statistically significant, while no significant interaction effects (group and group × time) were detected. ηp²: partial eta squared. Significant differences between groups indicated by ^*^P < 0.05 and ^**^P < 0.01.

In the BFR and BFR+BRJ groups, relative peak torque during CMJ testing for the left (Figure 4A) and right legs (Figure 4B) showed a significant main effect of time (*p* < 0.01), indicating post-intervention strength gains. For CMJ tests on the left, right, and both legs (Figure 4A,B), jump height, force, and RFD exhibited significant time effects (*p* < 0.001, partial η² > 0.06). Compared to pre-intervention, post-intervention CMJ performance improved significantly in both legs, with jump height and RFD increasing across conditions (*p* < 0.05 and *p* < 0.01, respectively). Force also increased, with a smaller improvement in the BFR group (*p* < 0.05, 95% CI [–272.40, –1.447]) and a larger improvement in the BFR+BRJ group (*p* < 0.01, 95% CI [−397.80, −35.24]). No significant differences were observed between the two groups. However, no significant changes in jump height (BFR group, pre: 22.13 ± 3.61 cm, post: 23.38 ± 3.75 cm; BFR+BRJ group, pre: 23.57 ± 4.72 cm, post: 24.12 ± 5.35 cm), force (BFR group, pre: 1502.69 ± 191.56 *N*, post: 1581.40 ± 168.80 N; BFR+BRJ group, pre: 1496.78 ± 220.82 *N*, post: 1551.72 ± 156.04 *N*), or RFD (BFR group, pre: 1745.34 ± 520.41 N/s, post: 1808.53 ± 460.59 N/s; BFR+BRJ group, pre: 1782.75 ± 554.60 N/s, post: 1868.69 ± 658.31 N/s) were observed in the left or right single-leg CMJ tests pre- to post-intervention in either group. These findings suggest that both BFR+BRJ and BFR interventions enhance bilateral jump performance but have no significant effect on single-leg performance.

### 3.4 Characteristics of fatigue-induced torque decline by 100 knee extensions at 90°/s

**Figure 5.**
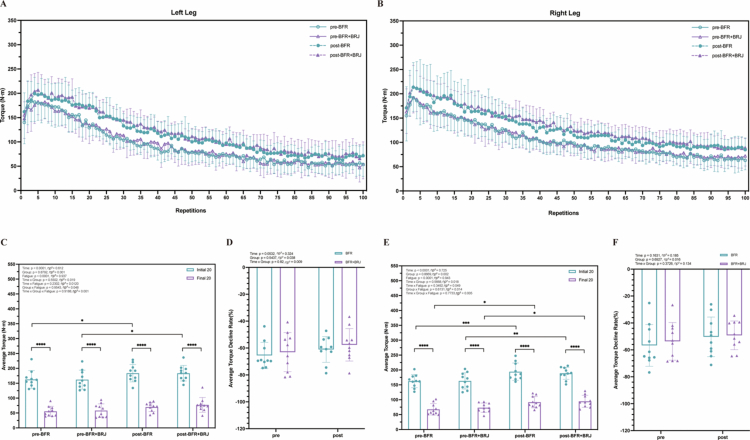
Comparison of Pre- and Post-Intervention Effects of BFR and BFR+BRJ on 100-Repetition Isometric Knee Extensor Maximal Voluntary Contraction Test. Torque values of knee extensors over 100 repetitions, and the non-fatigued (NF) average torque (initial 20) and fatigued (F) average torque (initial 20) at 90°/s. Values are presented as mean ± standard deviation (SD). Statistical analysis was conducted using a three-way repeated-measures ANOVA (group × fatigue × time) with Bonferroni's multiple comparisons test for post hoc analysis. Main effects for time (pre vs. post) and fatigue (NF vs. F) were statistically significant, while no significant interaction effects (group, group × time, group × fatigue, time × fatigue, or group × fatigue × time) were detected. Statistical significance levels are: ^*^P < 0.05, ^**^P < 0.01, ^***^P < 0.001 and ^****^P < 0.0001.

Changes in strength during 100 knee extensions at 90°/s are shown in Figure 4A,B. For both the left (Figure 5C) and right (Figure 5E) legs in the BFR and BFR+BRJ groups, the initial and final 20 repetitions’ average torque showed significant time effects (*p* < 0.0001, partial η² > 0.14) and fatigue effects (*p* < 0.0001, partial η² > 0.14). In both groups, average torque during the final 20 repetitions decreased significantly compared to the initial 20 repetitions (*p* < 0.0001), reflecting fatigue-induced strength decline in the knee extensors. Post-intervention, the initial 20 repetitions’ average torque in both the left and right legs significantly increased compared to pre-intervention (BFR: *p* < 0.05, left 95% CI [–40.72, –5.76], right 95% CI [–38.59, –4.28]; BFR+BRJ: *p* < 0.01, left 95% CI [–59.67, –9.68], right 95% CI [–53.06, –10.20]), and the final 20 repetitions’ average torque of the right leg also increased significantly in both groups (*p* < 0.05). These results suggest that both BFR+BRJ and BFR interventions improved knee extensor strength in a non-fatigued state and mitigated strength decline under fatigue. The fatigue-induced average torque decline rates in both the left (Figure 5D) and right legs (Figure 5F) were not significantly different pre- and post-intervention (>40%, Figure 5D,F), indicating that the fatigue protocol remained effective in inducing knee extensor fatigue before and after the intervention.

### 3.5 Intervention-induced changes in jump height, force and rate of force development (RFD) in CMJ test under fatigued vs. non-fatigued conditions

**Figure 6.**
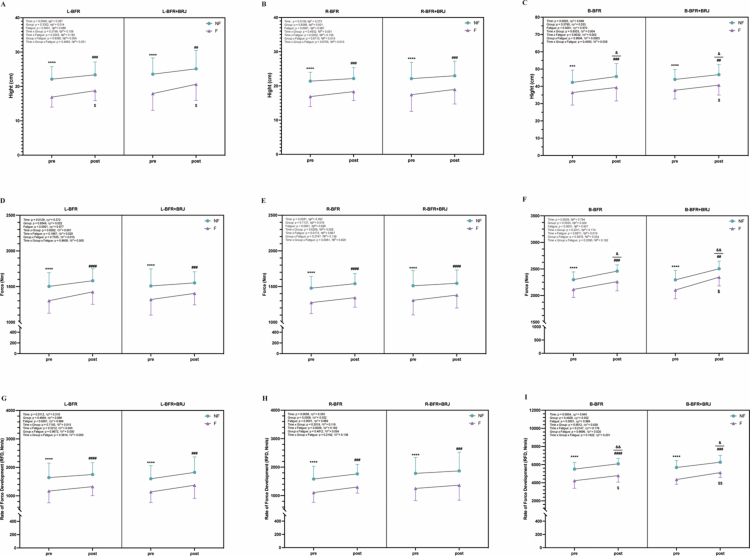
Movement Jump Test: Hight, Force and Rate of Force Development across Fatigued (F) and Non-Fatigued (NF) States (pre- and post-Intervention). Values are presented as mean ± standard deviation (SD). Significant differences between NF-pre and NF-post are represented by ^**&**^, and between F-pre and F-post by ^**$**^. Differences between pre-NF and pre-F are represented by ^*^, while those between post-NF and post-F are represented by ^#^. Statistical analysis was conducted using a three-way repeated-measures ANOVA (group × fatigue × time) with Bonferroni's multiple comparisons test for post hoc analysis. Main effects for time (pre vs. post) and fatigue (NF vs. F) were statistically significant, while no significant interaction effects (group, group × time, group × fatigue, time × fatigue, or group × fatigue × time) were detected. Statistical significance levels are: ^*^P < 0.05, ^**^P < 0.01, ^***^P < 0.001 and ^****^P < 0.0001.

Figure 5 shows the pre- and post-intervention changes in jump height, force, and RFD for the left (Figure 6A,D,G), right (Figure 6B,E,H), and both legs (Figure 6C,F,I) in the BFR and BFR+BRJ groups. Significant time effects (pre- vs. post-intervention, *p* < 0.05, partial η² > 0.06) and fatigue effects (non-fatigued vs. fatigued, *p* < 0.001, partial η² > 0.06) were observed. Pre-intervention fatigued left (BFR: *p* < 0.0001, 95% CI [–6.85, –3.6]; BFR+BRJ: *p* < 0.0001, 95% CI [–7.59, –3.68], Figure 6A), right (BFR: *p* < 0.0001, 95% CI [–6.33, –2.68]; BFR+BRJ: *p* < 0.001, 95% CI [–6.93, –2.44], Figure 6B), and both legs (BFR: *p* < 0.001, 95% CI [–8.76, –3.03]; BFR+BRJ: *p* < 0.001, 95% CI [–8.66, –3.28], Figure 6C) showed significantly reduced jump height compared to non-fatigued conditions. Post-intervention, the decline in fatigued jump height was improved in both groups’ left (BFR: *p* < 0.001, 95% CI [–6.69, –2.54]; BFR+BRJ: *p* < 0.01, 95% CI [–7.55, –1.44], Figure 6A), right (BFR: *p* < 0.001, 95% CI [–5.38, –2.27]; BFR+BRJ: *p* < 0.01, 95% CI [–6.32, –1.66], Figure 6B), and both legs (BFR: *p* < 0.001, 95% CI [–8.60, –4.04]; BFR+BRJ: *P* < 0.05, 95% CI [–10.71, –1.40], Figure 6C). Post-intervention, fatigued jump height increased significant at *p* < 0.05 across conditions in the left legs of both groups and in both legs of the BFR+BRJ group (Figure 6A,C). Force and RFD exhibited trends similar to jump height. Although post-intervention, fatigued left (BFR: *p* < 0.0001, 95% CI [–220.7, –90.64]; BFR+BRJ: *p* < 0.001, 95% CI [–223.00, –72.34], Figure 6D,G), right (BFR: *p* < 0.001, 95% CI [-226.40, -166.2]; BFR+ BRJ: *p* < 0.001, 95% CI [–195.59, –134.90], Figure 6E,H), and both legs (BFR: *p* < 0.001, 95% CI [–334.00, –63.07]; BFR+BRJ: *p* < 0.01, 95% CI [–238.70, –44.31], Figure 6F,I) still showed significant declines compared to non-fatigued conditions, the magnitude of decline was reduced compared to pre-intervention. Additionally, RFD in both legs significantly increased post-intervention in both groups under both non-fatigued (BFR: *p* < 0.01; BFR+BRJ: *p* < 0.05, Figure 6I) and fatigued (BFR: *p* < 0.05; BFR+BRJ: *p* < 0.01, Figure 6I) conditions.

### 3.6 Effects of the intervention on fatigue-induced rate of decline in jump height, force, and RFD

**Figure 7.**
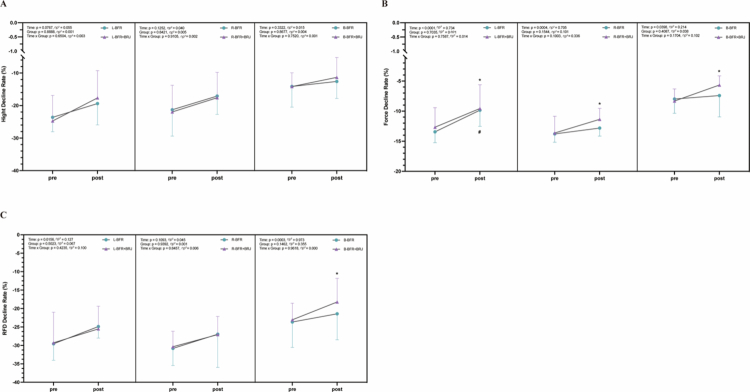
Pre- and Post-Intervention Changes in Decline Rate of Hight, Force and Rate of Force Development. Values are presented as mean ± standard deviation (SD). Significant differences between pre- and post-intervention are indicated by ^#^ for the BFR group and ^*^ for the BFR+BRJ group (^*^P < 0.05, ^**^P < 0.01 and ^***^P < 0.001).

Pre- and post-intervention, there were no significant differences in the height decline rate of the left, right and both legs in the BFR and BFR+BRJ groups during the fatigued CMJ test (Figure 7A). For the left, right, and both legs in the BFR+BRJ groups (Figure 7B), the force decline rate showed a significant time effect (*p* < 0.05, partial η² > 0.14). Post-intervention, the force decline rate of the left (*p* < 0.05, 95% CI [–5.57, –0.63]), right (*p* < 0.05, 95% CI [–4.07, –0.47]) and both legs (*p* < 0.05, 95% CI [–4.63, –0.68]) in the BFR+BRJ group significantly decreased, and the left leg in the BFR group also showed a similar downward trend (*p* < 0.05, 95% CI [–6.77, –0.42]). Furthermore, the RFD decline rate in the BFR+BRJ group only showed a significant decrease in both legs (*p* < 0.05, 95% CI [–8.44, –1.21], Figure 7C).

## 4 Discussion

This study primarily investigated the synergistic effects of BRJ supplementation on BFR training in enhancing lower-limb muscle strength and jump performance under fatigued and non-fatigued conditions. Four weeks of isokinetic BFR training significantly increased relative peak torque of knee flexors and extensors at 60°/s, 180°/s, and MVIC, confirming the effectiveness of BFR in improving muscle strength [19]. Additionally, BRJ supplementation enhanced knee extensor strength at 60°/s, likely due to elevated plasma nitrite levels following intake. Previous studies in humans [48,49] and rodents [31] have demonstrated that chronic dietary nitrate supplementation elevates NO levels, enhancing Ca²⁺ release from the sarcoplasmic reticulum [31] and optimizing excitation-contraction coupling [50], thereby improving neuromuscular function and increasing muscle contractility, particularly in type II fibers. Notably, CMJ performance has been widely used to assess neuromuscular status, lower-body power, and strength in athletes [8]. Current research indicates that BFR combined with lower-body strength training effectively enhances CMJ height, peak power, and RFD by improving muscle activation and potentiating PAP [20,22]. In this study, four weeks of BFR improved bilateral CMJ height, force, and RFD, while single-leg CMJ performance remained unchanged (*p* < 0.05). Previous studies showed that BRJ supplementation alone did not significantly improve CMJ height [35,36]. Similarly, this study found that BRJ combined with BFR training did not further enhance non-fatigued CMJ height but resulted in greater improvements in CMJ force (*p* < 0.01), highlighting its effect on muscle strength rather than jump height.

In sports requiring frequent jumping, continuous jumping induces lower-limb neuromuscular fatigue, which can impair performance and increase the risk of injury [3,7]. In this study, a 100-repetition isometric knee extensor maximal voluntary contraction test was used to induce lower-body muscle fatigue, simulating post-fatigue lower-limb performance. Post-intervention, both groups showed significant improvements in average torque during non-fatigued (initial 20) and fatigued (final 20) repetitions, indicating that BFR enhances fatigued muscle strength. It is noteworthy that chronic BRJ supplementation may enhance lower-limb muscle fatigue resistance and CMJ performance. Compared to pre-intervention, BRJ supplementation further supported post-fatigue CMJ performance, reducing fatigue-related declines in jump height, force, and RFD. This suggests that BRJ supplementation may enhance fatigue resistance alongside BFR-induced improvements in lower-limb muscle strength, thus mitigating fatigue-related performance decline during exercise. Research by Daab et al. demonstrated that chronic BRJ supplementation (8 mmol/L nitrate for 7 consecutive days) significantly alleviated neuromuscular fatigue during simulated soccer matches [33]. Compared to the control group, BRJ supplementation significantly reduced the decline in maximal voluntary contraction capacity and mitigated both central and peripheral fatigue. Additionally, in female volleyball players, BRJ supplementation did not directly enhance CMJ height but indirectly supported muscle fatigue recovery by reducing muscle swelling and soreness [38]. Physiologically, BRJ may have synergistic effects with the local hypoxic conditions induced by BFR training. Skeletal muscle serves as a major site for nitrate storage and metabolism in humans [51–53]. It has been proposed that under conditions of low oxygen, such as during BFR-induced hypoxia, stored nitrate may be reduced to nitrite via nitrate reductase pathways, potentially contributing to nitric oxide (NO) production, as suggested by animal and in vitro studies [52,54,55]. NO-induced vasodilation enhances energy delivery and lactate clearance, thereby amplifying metabolic responses following BFR-induced hypoxia. This may improve muscle cell oxygen utilization and energy production rates by modulating mitochondrial respiratory chain complexes and mitochondrial biogenesis in skeletal muscle [56,57]. Notably, hypoxic conditions have been shown to further enhance nitrite-to-NO conversion, increasing skeletal muscle nitrate utilization and NO production during BFR, thereby triggering more pronounced metabolic responses [51,58]. Thus, the combination of BFR and BRJ likely promotes both strength gains and fatigue resistance, offering practical benefits for training and sport performance.

In summary, the findings provide a theoretical foundation for optimizing athletes’ training methods and improving jumping performance through BFR training and BRJ supplementation strategies. However, some limitations of this study should be acknowledged when interpreting and applying the results. First, the study participants were limited to male students majoring in physical education, which constrains the generalizability of the findings. Second, the lack of systematic and standardized control over participants' daily dietary patterns and caloric intake necessitates consideration of potential confounding effects from dietary differences. Moreover, as real-time monitoring of participants’ daily habits during the intervention was not possible, we relied on verbal instructions and periodic reminders to encourage adherence to the study guidelines. Although no participants reported adverse effects from BRJ supplementation, the absence of detailed dietary logs suggests that potential risks associated with nitrate intake should be monitored in future research [59]. In addition, training-load monitoring variables, such as ratings of perceived exertion and discomfort during BFR sessions, were not collected, which may influence the interpretation of the training stimulus and participant tolerance. Finally, this study did not assess mechanistic biomarkers, including oxidative stress and inflammatory markers, which limits understanding of the antioxidant and metabolic effects of BRJ supplementation. Future studies including such measures are warranted to clarify the molecular pathways underlying the synergistic effects of BFR and BRJ.

## Artificial intelligence (AI)

All content in this manuscript was written and proofread solely by the author. ChatGPT (GPT-3.5) was used only for grammar checking and polishing.
